# Early impact of 13-valent pneumococcal conjugate vaccine on pneumococcal meningitis—Burkina Faso, 2014–2015

**DOI:** 10.1016/j.jinf.2017.12.002

**Published:** 2018-03

**Authors:** Dinanibè Kambiré, Heidi M. Soeters, Rasmata Ouédraogo-Traoré, Isaïe Medah, Lassana Sangaré, Issaka Yaméogo, Guetawendé Sawadogo, Abdoul-Salam Ouédraogo, Soumeya Ouangraoua, Lesley McGee, Velusamy Srinivasan, Flavien Aké, Malika Congo-Ouédraogo, Absatou Ky Ba, Cynthia G. Whitney, Ryan T. Novak, Chris Van Beneden

**Affiliations:** a Centre Hospitalier Universitaire Pédiatrique Charles De Gaulle, Ouagadougou, Burkina Faso; b Epidemic Intelligence Service, Centers for Disease Control and Prevention (CDC), Atlanta, GA, USA; c National Center for Immunization and Respiratory Diseases, CDC, Atlanta, GA, USA; d Ministère de la Santé, Ouagadougou, Burkina Faso; e Centre Hospitalier Universitaire-Yalgado Ouédraogo, Ouagadougou, Burkina Faso; f Centre Hospitalier Universitaire Sourou Sanou, Bobo-Dioulasso, Burkina Faso; g Centre Muraz, Bobo-Dioulasso, Burkina Faso; h Davycas International, Ouagadougou, Burkina Faso; i Laboratoire National de Santé Publique, Ouagadougou, Burkina Faso

**Keywords:** Pneumococcal conjugate vaccine, Meningitis, Pneumococcal meningitis, Surveillance, Vaccine impact, Burkina Faso

## Abstract

**Objectives:**

We evaluate early impact of 13-valent pneumococcal conjugate vaccine (PCV13) on pneumococcal meningitis in Burkina Faso.

**Methods:**

Nationwide surveillance gathered demographic/clinical information and cerebrospinal fluid (CSF) results for meningitis cases. Pneumococcal cases were confirmed by culture, polymerase chain reaction (PCR), or latex agglutination, and strains serotyped using PCR. We compared incidence (cases per 100,000) in the early post-PCV13 period (2014 and 2015) to average pre-PCV13 incidence (2011–2013).

**Results:**

In 2015, age-specific pneumococcal meningitis incidences were 8.7 (<1 year), 2.4 (1–4 years), 6.5 (5–14 years), and 2.6 (≥15 years). Compared to 2011–2013, PCV13-serotype incidence among all ages decreased by 32% (95%CI: 23%–39%), with significant decreases among children aged <1 year (76%; 95%CI: 64%–84%) and 1–4 years (58%, 95%CI: 40%–71%). Among all ages, incidence of PCV13 serotypes besides serotype 1 decreased (68%; 95%CI: 59%–75%), but serotype 1 incidence did not. Incidence of non-PCV13 serotypes also decreased (47%; 95%CI: 29%–60%). Among children aged <1 year, serotypes 12F/12A/12B/44/46 (17%), 1 (12%), and 5 (10%) predominated.

**Conclusions:**

Following PCV13 introduction, PCV13-serotype meningitis incidence in young children significantly decreased. PCV13 impact on serotype 1 and disease in older children and adults requires continued monitoring.

## Highlights

• Burkina Faso introduced 13-valent pneumococcal conjugate vaccine (PCV13) in 2013.
• By 2015, incidence of PCV13 pneumococcal meningitis decreased by 32%.
• Large decreases occurred among children aged <1 year (76%) and 1–4 years (58%).

## Introduction

*Streptococcus pneumoniae* is a leading infectious cause of global morbidity and mortality^1^ and primary etiology of bacterial meningitis, along with *Neisseria meningitidis* and *Haemophilus influenzae* type b (Hib).^2^ In the meningitis belt of sub-Saharan Africa,^3^ pneumococcal meningitis has a distinct seasonality similar to that of meningococcal meningitis, high case fatality ratio (CFR), and predominance of serotype 1 disease in persons aged >5 years.^4^, ^5^, ^6^, ^7^

Burkina Faso, a West African country located entirely within the meningitis belt, experiences hyper-endemic rates of meningitis.^8^ Historically, most meningitis cases were due to *N. meningitidis* serogroup A.^3^ Following the successful introductions of Hib vaccine in 2006 and MenAfriVac^TM^ in 2010,^3^ *S. pneumoniae* became the primary bacterial meningitis pathogen. In 2013, Burkina Faso introduced 13-valent pneumococcal conjugate vaccine (PCV13) into the routine infant immunization program.

To date, 55 Gavi-supported countries have introduced pneumococcal conjugate vaccines (PCVs) into routine immunization programs.^9^ To help sustain these programs, evaluating PCV impact on pneumococcal disease and circulating serotypes is key. PCV introduction resulted in substantial decreases in invasive pneumococcal disease (IPD) and pneumonia among children globally, with most dramatic decreases observed among young children targeted for vaccination.^10^, ^11^, ^12^, ^13^, ^14^, ^15^, ^16^ In addition to direct effects on disease, widespread use of PCVs can also reduce nasopharyngeal carriage of vaccine-type pneumococci among both vaccinated and unvaccinated individuals.^11^ The resulting herd protection decreases PCV-type IPD incidence in unvaccinated children and adults.^17^, ^18^ Data on PCV impact in Asia and West Africa are limited.^14^ It is important to evaluate whether PCV delivered on the World Health Organization (WHO) Expanded Program on Immunization routine schedule of three early-infancy doses can control disease in high-transmission settings, such as meningitis belt countries, as effectively as PCV programs in other settings.

We previously described case-based meningitis surveillance data prior to PCV13 introduction (2011–2013) in Burkina Faso – one of the few African countries to successfully implement case-based surveillance nationwide.^19^ The highest pneumococcal meningitis incidence and mortality occurred among children aged <1 year, and 71% of cases were due to PCV13 serotypes. Here, we evaluate early PCV13 impact by comparing pneumococcal meningitis surveillance data from before (2011–2013) and after (2014 and 2015) PCV13 introduction in Burkina Faso, thereby helping fill the information gap on PCV impact in West Africa.

## Methods

### PCV13 vaccination

PCV13 was introduced into the routine immunization program nationwide on October 31, 2013, with doses administered to children aged 8, 12, and 16 weeks. No catch-up doses were recommended for older children.

### National surveillance system

Burkina Faso has collected high-quality case-based meningitis surveillance data since 2010, and joined MenAfriNet in 2015.^19^, ^20^ Case-level demographic and clinical information, as well as cerebrospinal fluid (CSF) specimens, are collected from all suspect meningitis cases in all 63 districts using WHO and MenAfriNet instruments^19^, ^21^ and tested at five national reference laboratories.

According to WHO case definitions,^22^ a suspected meningitis case has sudden onset of fever ≥38.5 °C with neck stiffness, altered consciousness, or other meningeal signs (including flaccid neck, bulging fontanel, or convulsions in young children). A laboratory-confirmed pneumococcal meningitis case is a suspected case with *S. pneumoniae* isolated from CSF by culture or detected in CSF by real-time polymerase chain reaction (rt-PCR) or latex agglutination using laboratory methods previously described.^19^ Single-target rt-PCR was unable to differentiate some genetically similar serotypes (e.g., 12F/12A/12B/44/46).

### PCV13 vaccination status among PCV13-eligible children

Vaccination records were sought for children with pneumococcal meningitis born after August 1, 2013 and therefore potentially eligible to receive PCV13. As PCV13 vaccination status is not routinely reported, the reporting districts were asked to retrospectively abstract the case child's local vaccination records, using a standardized procedure and data collection tool.

### Statistical methods

We analyzed meningitis cases diagnosed from January 1, 2011 to December 31, 2015. A pre-PCV13 period (2011–2013) and a post-PCV13 period (2014 and 2015) were defined. Cases in non-residents of Burkina Faso were excluded from analyses.

Administrative PCV13 coverage was calculated by dividing the reported number of administered doses by population estimates for children aged 0–11 months projected from the 2006 national census. As PCV13 is sometimes given to children outside the eligible age range and population denominators are estimated, administrative coverage can exceed 100%. In 2015, Burkina Faso had a total population of 18,450,494 and an estimated birth cohort of 732,675.

Pneumococcal meningitis cases were categorized as due to PCV13 serotypes (1, 3, 4, 5, 6A/6B, 7F/7A, 9V/9A, 14, 18C/18F/18B/18A, 19A, 19F, or 23F), non-PCV13 serotypes, or non-typeable strains. Annual incidences (cases per 100,000 persons) were calculated using projected age-stratified population census estimates. Within each age stratum (<1 years, 1–4 years, 5–9 years, 10–14 years, and ≥15 years), the number of cases confirmed by culture or rt-PCR as *S. pneumoniae* was divided by the number of cases with CSF tested via culture or rt-PCR at a national laboratory. This proportion was then applied to the number of suspected meningitis cases within that age stratum for which no diagnostic testing was performed; this number was then added to confirmed cases to calculate the adjusted incidence. Comparisons of the characteristics of suspected meningitis cases that were tested vs. not tested at a national laboratory in Burkina Faso from 2011–2015 have been previously described.^20^

To calculate the incidence of PCV13, non-PCV13, and non-typeable pneumococcal meningitis, the adjusted pneumococcal meningitis incidence in each age group was multiplied by the proportion of serotyped cases in each category. Percentage change ([relative risk − 1] × 100) in incidence with 95% confidence intervals (95%CI) was calculated using the mean incidence pre-PCV13 (2011–2013) and each year post-PCV13 (2014 and 2015) and the Poisson distribution for incidence rates. CFRs were calculated by dividing the number of reported deaths by the total number of cases.

### Study approval

This analysis was approved by Burkina Faso Ministry of Health ethical committee and was determined by the Centers for Disease Control and Prevention's Human Research Protection Office to be public health non-research.

### Role of the funding source

The funders of the study had no role in study design, data collection, data analysis, data interpretation, or writing of the report. The corresponding authors had full access to all the data in the study and had final responsibility for the decision to submit for publication.

## Results

### PCV13 vaccine coverage

By the end of 2015, administrative coverage of the first, second, and third PCV13 doses was 108%, 103%, and 105%, respectively (Supplementary Fig. S1).

### Data completeness and quality

From 2011–2015, 18,538 suspected meningitis cases were reported; nearly all (97%) had CSF collected (Supplementary Table S1). Ninety percent of CSF specimens were tested by Gram stain, 22% by latex, 22% by culture, and 46% by rt-PCR. In total, 61% were tested by latex, culture, or rt-PCR. The percentage of all CSFs tested via culture or rt-PCR at a national laboratory increased from 45% in 2011 to 74% in 2015.

*S. pneumoniae* detection varied by diagnostic test (Supplementary Table S2). In 2014–2015, 318 laboratory-confirmed pneumococcal meningitis cases were tested using latex, of which 275 (86.5%) were positive for *S. pneumoniae*; comparatively, 46 (22.6%) of 204 tested by culture grew *S. pneumoniae*. The majority (984/1053; 93.4%) of pneumococcal meningitis cases were confirmed using rt-PCR, with or without another positive test. Although testing practices varied by year, sensitivity of each of the three methods remained consistent.

### Pneumococcal meningitis surveillance data

From 2011–2015, 2581 meningitis cases were laboratory-confirmed via latex, culture, or rt-PCR as *S. pneumoniae*, with 557 (22%) deaths (Table 1). CFR was highest among infants and adults: 29% in children aged <1 year, 15% in ages 1–4 years, 19% in ages 5–9 years, 19% in ages 10–14 years, and 23% in ages ≥15 years.

**Table 1:** Pneumococcal meningitis cases, Burkina Faso, 2011–2015.

	2011		2012		2013		Pre-PCV13 2011–2013		2014		2015		Post-PCV13 2014–2015		Total	
	N (%)		N (%)		N (%)		N (%)		N (%)		N (%)		N (%)		N (%)	
Laboratory-confirmed pneumococcal meningitis cases^a^	642	(75)	462	(36)	424	(60)	1,528	(53)	502	(66)	551	(62)	1,053	(64)	2,581	(57)
Age group:																
<1 year	104	(16)	89	(19)	83	(20)	276	(18)	68	(14)	45	(8)	113	(11)	389	(15)
1 year	19	(3)	25	(5)	21	(5)	65	(4)	17	(3)	9	(2)	26	(2)	91	(4)
2–4 years	54	(8)	46	(10)	37	(9)	137	(9)	34	(7)	39	(7)	73	(7)	210	(8)
5–9 years	134	(21)	97	(21)	81	(19)	312	(20)	113	(23)	141	(26)	254	(24)	566	(22)
10–14 years	125	(20)	81	(18)	81	(19)	287	(19)	113	(23)	124	(23)	237	(23)	524	(20)
15–29 years	114	(18)	68	(15)	71	(17)	253	(17)	83	(17)	120	(22)	203	(19)	456	(18)
≥30 years	92	(14)	56	(12)	50	(12)	198	(13)	74	(15)	73	(13)	147	(14)	345	(13)
Reported deaths	179	(28)	94	(20)	84	(20)	357	(23)	99	(20)	101	(18)	200	(19)	557	(22)

a *S. pneumoniae* isolated from CSF by culture or detected in CSF by rt-PCR or latex.

The proportion of pneumococcal meningitis cases among all ages that were serotyped increased from 68% in 2011–2013 to 83% in 2014–2015 (Table 2, Supplementary Table S3). In 2014–2015, 78 of 1053 cases were only positive via latex and 98 culture- or rt-PCR-positive cases did not have a specimen or isolate available for serotyping, leaving 877 (83%) specimens that could be serotyped. Among these, 638 (73%) were PCV13 serotypes, 100 (11%) were non-PCV13 serotypes, and 139 (16%) were non-typeable, similar to the 71%, 14%, and 15%, respectively, among 1036 serotyped specimens in 2011–2013 (P = 0.3).

**Table 2:** Distribution of pneumococcal serotypes by age, Burkina Faso, 2014–2015.

Pneumococcal serotype	<1 year N (%)		1 year N (%)		2 years N (%)		3–4 years N (%)		≥5 years N (%)		Total N (%)	
*PCV13 serotypes*	*49*	*(54)*	*14*	*(70)*	*15*	*(71)*	*27*	*(66)*	*533*	*(76)*	*638*	*(73)*
1	11	(12)	5	(25)	7	(33)	15	(37)	450	(64)	488	(56)
3	1	(1)	0	(0)	0	(0)	0	(0)	10	(1)	11	(1)
4	1	(1)	0	(0)	0	(0)	0	(0)	7	(1)	8	(1)
5	9	(10)	0	(0)	0	(0)	1	(2)	16	(2)	26	(3)
6A/6B	7	(7)	1	(5)	3	(14)	3	(7)	11	(2)	25	(3)
7F/7A	4	(4)	0	(0)	0	(0)	0	(0)	2	(0.3)	6	(1)
9V/9A	0	(0)	1	(5)	1	(5)	1	(2)	4	(1)	7	(1)
14	5	(5)	1	(5)	0	(0)	0	(0)	9	(1)	15	(2)
18C/18F/18B/18A	1	(1)	2	(10)	0	(0)	1	(2)	4	(1)	8	(1)
19A	0	(0)	0	(0)	1	(5)	0	(0)	2	(0.3)	3	(0.3)
19F	2	(2)	2	(10)	0	(0)	1	(2)	6	(1)	11	(1)
23F	8	(9)	2	(10)	3	(14)	5	(12)	12	(2)	30	(3)
*Non-PCV13 serotypes*	*23*	*(25)*	*1*	*(5)*	*1*	*(5)*	*3*	*(7)*	*72*	*(10)*	*100*	*(11)*
2	4	(4)	0	(0)	0	(0)	1	(2)	5	(1)	10	(1)
7C/7B/40	1	(1)	0	(0)	0	(0)	0	(0)	0	(0)	1	(0.1)
9N/9L	1	(1)	0	(0)	0	(0)	0	(0)	2	(0.3)	6	(1)
10A	0	(0)	0	(0)	0	(0)	0	(0)	1	(0.1)	1	(0.1)
10F/10C/33C	0	(0)	0	(0)	0	(0)	0	(0)	1	(0.1)	1	(0.1)
11A/11D	1	(1)	0	(0)	0	(0)	0	(0)	5	(1)	6	(1)
12F/12A/12B/44/46	15	(17)	0	(0)	1	(5)	2	(5)	44	(6)	62	(7)
13	0	(0)	0	(0)	0	(0)	0	(0)	1	(0.1)	1	(0.1)
15A/15F	0	(0)	0	(0)	0	(0)	0	(0)	1	(0.1)	1	(0.1)
15B/15C	1	(1)	0	(0)	0	(0)	0	(0)	1	(0.1)	2	(0.2)
16F	0	(0)	0	(0)	0	(0)	0	(0)	1	(0.1)	1	(0.1)
23B	0	(0)	0	(0)	0	(0)	0	(0)	1	(0.1)	1	(0.1)
25F/25A/38	0	(0)	0	(0)	0	(0)	0	(0)	9	(1)	9	(1)
35B	0	(0)	1	(5)	0	(0)	0	(0)	0	(0)	1	(0.1)
*Non-typeable serotypes*	19	(21)	5	(25)	5	(24)	11	(27)	99	(14)	139	(16)
Total serotyped	91	(81)	20	(77)	21	(87)	41	(84)	704	(84)	877	(83)
Missing serotype^a^	22	(19)	6	(23)	3	(13)	8	(16)	137	(16)	176	(17)
Total	113		26		24		49		841		1053	

a 78 cases were only positive via latex and could not be serotyped: 50 from 2014 and 28 from 2015. Serotype results were unavailable for 98 culture- and/or rt-PCR-positive cases: 62 from 2014 and 36 from 2015.

**Fig. 1:**
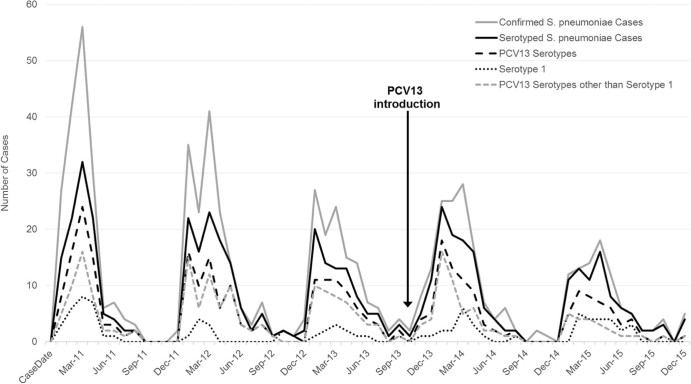
Epidemic curve of confirmed pneumococcal meningitis cases, serotyped cases, PCV13 serotypes, serotype 1, and PCV13 serotypes other than serotype 1 among children aged <5 years, by month, Burkina Faso, 2011–2015. *S. pneumoniae* isolated from cerebrospinal fluid (CSF) by culture or detected in CSF by real-time polymerase chain reaction or latex.

In 2014–2015, the predominant serotypes by age group were: 12F/12A/12B/44/46 (17%), 1 (12%), and 5 (10%) among children aged <1 year; 1 (33%), 23F (12%), and 6A/6B (9%) among ages 1–4 years; and 1 (64%) and 12F/12A/12B/44/46 (6%) in persons aged ≥5 years (Table 2). The proportion of cases due to serotype 1 increased with increasing age, from 12% in children aged <1 year to 64% in those persons ≥5 years. Among children aged <5 years, 61% of cases were due to PCV13 serotypes; 39% were due to PCV13 serotypes other than serotype 1 (Fig. 1). Serotype 1 was responsible for 76% of PCV13 cases among all ages in 2014–2015, compared to 63% in 2011–2013 (Supplementary Table S3).

### PCV13 impact

**Fig. 2:**
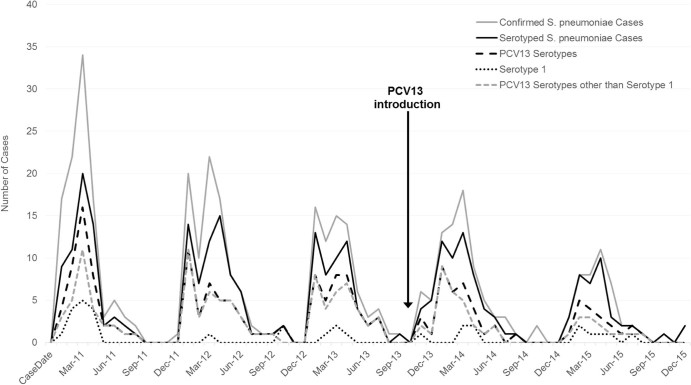
Epidemic curve of confirmed pneumococcal meningitis cases, serotyped cases, PCV13 serotypes, serotype 1, and PCV13 serotypes other than serotype 1 among children aged <1 year, by month, Burkina Faso, 2011–2015. *S. pneumoniae* isolated from cerebrospinal fluid (CSF) by culture or detected in CSF by real-time polymerase chain reaction or latex.

From pre-PCV13 baseline (2011–2013) to 2015, pneumococcal meningitis incidence among all ages decreased by 30% (95%CI: 24%–36%) from 5.6 to 3.9 cases per 100,000 (Table 3). The largest declines were observed among children aged <1 year (68%; 95%CI: 57%–76%; 26.9 to 8.7) and 1–4 years (55%; 95%CI: 39%–66%; 5.4 to 2.4) (Table 3, Fig. 2). No significant change was observed among persons aged ≥5 years (Table 3, Supplementary Fig. S2).

**Table 3:** Annual incidence (cases per 100,000 persons) of pneumococcal meningitis^a^^,^^b^, Burkina Faso, 2011–2015.

	2011	2012	2013	Pre-PCV13 (2011–2013)	2014	2015	Percentage change (95% Confidence Interval)	
							2014 only vs. 2011–2013	2015 only vs. 2011–2013
All pneumococcal meningitis cases	7.1	6.2	3.5	5.6	4.1	3.9	−27% (−33%, −29%)	−30% (−36%, −24%)
<1 year	31.3	32.9	16.5	26.9	13.7	8.7	−49% (−60%, −35%)	−68% (−76%, −57%)
1–4 years	6.6	6.5	3.1	5.4	3.2	2.4	−41% (−55%, −23%)	−55% (−66%, −39%)
5–14 years	9.4	7.7	4.4	7.2	6.2	6.5	−14% (−26%, 0%)	−10% (−22%, 4%)
≥15 years	4.0	3.1	2.0	3.0	2.5	2.6	−19% (−38%, −4%)	−16% (−29%, 0%)
PCV13 serotypes	5.3	4.4	2.2	4.0	3.1	2.7	−22% (−30%, −13%)	−32% (−39%, −23%)
<1 year	21.7	19.1	11.2	17.3	7.9	4.2	−55% (−67%, −38%)	−76% (−84%, −64%)
1–4 years	4.6	4.3	2.0	3.6	2.4	1.5	−35% (−53%, −10%)	−58% (−71%, −40%)
5–14 years	7.3	5.8	2.8	5.3	4.8	4.5	−9% (−23%, 8%)	−14% (−28%, 2%)
≥15 years	2.9	2.3	1.2	2.1	1.9	1.7	−13% (−29%, 7%)	−20% (−35%, −1%)
Non-PCV13 serotypes	0.5	0.9	0.7	0.7	0.6	0.4	−22% (−40%, 1%)	−47% (−60%, −29%)
<1 year	2.5	8.3	4.0	5.0	4.2	1.6	−15% (−48%, 38%)	−69% (−84%, −39%)
1–4 years	0.3	1.0	0.5	0.6	0.3	0.1	−48% (−78%, 21%)	−90% (−92%, −50%)
5–14 years	0.9	1.0	0.9	0.9	0.7	0.8	−27% (−53%, 12%)	−17% (−45%, 26%)
≥15 years	0.4	0.4	0.5	0.4	0.2	0.4	−50% (−71%, −15%)	−6% (−39%, 47%)
Non-typeable serotypes	1.2	0.8	0.6	0.9	0.4	0.8	−52% (−64%, −37%)	−6% (−25%, 18%)
<1 year	7.1	5.4	1.3	4.6	1.6	2.9	−66% (−83%, −33%)	−37% (−64%, 9%)
1–4 years	1.7	1.1	0.6	1.2	0.5	0.9	−59% (−79%, −20%)	−25% (−56%, 30%)
5–14 years	1.2	0.9	0.7	0.9	0.7	1.1	−29% (−54%, 10%)	23% (−16%, 80%)
≥15 years	0.7	0.4	0.4	0.5	0.4	0.4	−21% (−49%, 23%)	−7% (−39%, 41%)
PCV13 serotypes other than serotype 1	1.6	1.9	1.0	1.5	1.0	0.5	−36% (−47%, −23%)	−68% (−75%, −59%)
<1 year	14.6	17.7	9.8	14.0	6.6	2.9	−53% (−67%, −34%)	−79% (−87%, −67%)
1–4 years	2.7	3.2	1.4	2.4	1.5	0.6	−37% (−58%, −6%)	−76% (−86%, −58%)
5–14 years	1.2	1.2	0.8	1.1	0.9	0.6	−17% (−44%, 23%)	−41% (−62%, −9%)
≥15 years	0.5	0.5	0.2	0.4	0.4	0.3	−8% (−42%, 46%)	−33% (−59%, 11%)
Serotype 1	3.7	2.5	1.2	2.5	2.2	2.2	−13% (−24%, 0%)	−10% (−21%, 4%)
<1 year	7.1	1.5	1.3	3.3	1.3	1.3	−60% (−81%, −15%)	−59% (−81%, −14%)
1–4 years	1.9	1.1	0.7	1.2	0.9	0.9	−30% (−59%, 21%)	−25% (−56%, 27%)
5–14 years	6.1	4.6	2.0	4.2	4.0	3.9	−7% (−23%, 13%)	−8% (−24%, 11%)
≥15 years	2.4	1.8	0.9	1.7	1.5	1.4	−14% (−34%, 9%)	−17% (−34%, 5%)

a *S. pneumoniae* isolated from cerebrospinal fluid (CSF) by culture or detected in CSF by real-time polymerase chain reaction or latex.

b Incidence adjusted for the proportion of cases with CSF tested at a national laboratory.

**Fig. 3:**
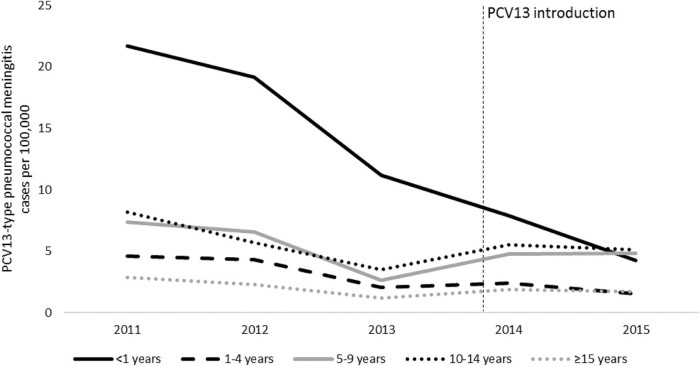
Incidence of pneumococcal meningitis caused by PCV13 serotypes, by year and age group, Burkina Faso, 2011–2015.

By 2015, incidence of PCV13 serotypes among all ages decreased by 32% (95%CI: 23%–39%), with significant decreases observed among children aged <1 year (76%; 95%CI: 64%–84%) and 1–4 years (58%; 95%CI: 40%–71%) (Table 3, Fig. 3). Incidence of non-PCV13 serotypes also declined: 47% (95%CI: 29%–60%) among all ages, 69% (95%CI: 39%–84%) among children aged <1 year, and 90% (95%CI: 50%–92%) among children aged 1–4 years (Supplementary Fig. S3). However, the absolute decline in incidence was larger for PCV13 serotypes than for non-PCV13 serotypes: 13.1 cases per 100,000 vs. 3.4 cases per 100,000 among children aged <1 year (and 1.3 cases per 100,000 vs. 0.3 cases per 100,000 among all ages). A decrease in incidence among all ages was observed for the twelve PCV13 serotypes other than serotype 1 (68%; 95%CI: 59%–75%), with significant declines among children aged <1 year, 1–4 years, and 5–14 years. However, serotype 1 only significantly decreased among children aged <1 year (59%; 95%CI: 14%–81%), with no significant change observed among all ages (10%; 95%CI: −4% to 21%). Incidence of non-typeable disease did not significantly change (6%; 95%CI: −18% to 25%).

**Fig. 4:**
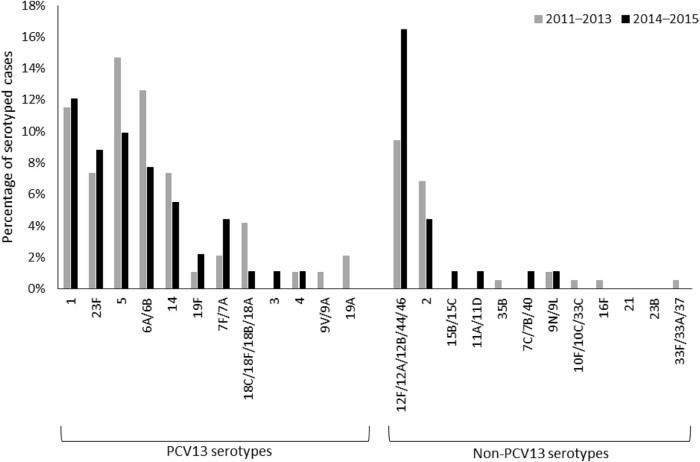
Percentage of serotyped pneumococcal meningitis cases due to each serotype among children aged <1 year in 2011–2013 vs. 2014–2015, Burkina Faso. Non-typeable serotypes are not shown in this figure.

Among children aged <1 year, the proportion of serotyped cases caused by serotypes 5, 6A/6B, and 18C/18F/18B/18A was lower in 2014–2015 than in 2011–2013, while the proportion of cases caused by serotypes 1, 23F, and 12F/12A/12B/44/46 increased (Fig. 4). Among all ages, the proportion of serotyped cases due to serotype 1 increased (Supplementary Fig. S4).

### PCV13 vaccination status among PCV13-eligible children

PCV13 vaccination records were located for 74 (94%) of 79 pneumococcal meningitis cases among PCV13-eligible children. Of these, 22 (30%) had not received a PCV13 dose ≥2 weeks prior to illness onset, 12 (16%) received 1 dose, 14 (19%) 2 doses, and 26 (35%) 3 doses. Fifty-seven (77%) had a known serotype; 26 (35%) were PCV13 serotypes. Nine children were infected with a PCV13 serotype despite receiving 3 doses of PCV13: 4 with serotype 23F, 3 with serotype 1, and 1 each with serotypes 5 and 6A/6B (Supplementary Table S4). Among children who received 2–3 doses, we found no significant difference in time since last dose for disease caused by serotype 1 (median: 5.4 months; range: 1.1–7.5), other PCV13 serotypes (median: 6.3; range: 1.2–7.9) or non-PCV13 serotypes (median: 6.0; range: 0.5–14.2) (Supplementary Table S5).

## Discussion

Our findings suggest an early direct effect of Burkina Faso's infant PCV13 program, as PCV13-serotype meningitis incidence among children aged <1 year was 76% lower in 2015 compared to pre-PCV13, equivalent to an absolute decline of 13.1 cases per 100,000. PCV13 impact appeared to be greater for PCV13 serotypes besides serotype 1 (79% decrease) than for serotype 1 (59% decrease) among this vaccine-eligible age group. While children aged 1–4 years experienced a smaller percentage decrease in PCV13-serotype incidence than in non-PCV13 serotype incidence (58% vs. 90%, respectively), they experienced a greater absolute decrease in PCV13-serotype incidence than in non-PCV13 serotype incidence (2.1 cases per 100,000 vs. 0.5 cases per 100,000, respectively). Whether the PCV13 program has indirect benefits – reducing disease among those too old to be vaccinated – was not yet clear from this early data.

The reason for the decline in non-PCV13 serotype incidence is unclear, though an increase in non-PCV13 serotype disease from 2011 to 2012 (2.5 to 8.3 cases per 100,000) drives this decline. A large meningococcal meningitis epidemic occurred in 2012,^23^ which doubled the annual number of suspected meningitis cases and may have increased healthcare-seeking behavior or collection and testing of CSF specimens. Environmental conditions driving the meningococcal epidemic could have also increased individual susceptibility to pneumococcal meningitis. Therefore, 2011 and 2013 are considered as more stable baseline years; comparing 2014–2015 to 2011/2013 reveals significant declines in PCV13 serotypes but not in non-PCV13 serotypes, while suspected, probable, meningococcal, and *H. influenzae* meningitis incidence remained stable.^20^ Alternatively, the years used as a pre-PCV13 baseline (2011–2013) could represent a natural high point in the cyclical nature of pneumococcal meningitis incidence. In the absence of more years of quality pre-PCV13 surveillance data, it is difficult to establish a true baseline.

Serotype 1 continues to dominate as a cause of pneumococcal meningitis following PCV13 introduction, causing 56% of pneumococcal meningitis in all age groups and 64% of cases among those aged ≥5 years. Similar to meningococcal serogroups, serotype 1 has natural variations in incidence over time and can cause outbreaks when population immunity wanes.^24^ It is difficult to know what the natural trend of serotype 1 in 2014–2015 would have been in the absence of PCV13 introduction. Also, serotype 1 transmission may differ from other serotypes, as it is not commonly found in pneumococcal carriage studies.^25^ As in The Gambia,^14^ these factors make it difficult to assess PCV13 impact on serotype 1, although our data clearly show that PCV13 is not yet controlling serotype 1 disease. PCV13 given on a compressed schedule (3 doses given at 1-month intervals) early in life may not be as effective against serotype 1 as it is against other PCV13 serotypes circulating in Burkina Faso. More birth cohorts may need to receive PCV13 or a booster dose may be needed^26^ before serotype 1 is better controlled.

In 2014–2015, 25% of pneumococcal meningitis infections among children aged <1 year were due to non-PCV13 serotypes. In particular, 65% of non-PCV13 serotypes were 12F/12A/12B/44/46, serotypes previously reported to have a high prevalence in the region and which may be considered for inclusion in future PCV formulations. Serotype 12F is emerging as an important cause of IPD with the propensity to cause outbreaks.^27^, ^28^ We also found a high proportion (16%) of non-typeable strains in 2014–2015, despite using both rt-PCR and conventional PCR methods. This is consistent with 2011–2013^19^ and with previous studies in the region,^7^ and may reflect circulating serotypes not covered by existing detection methods or low antigen levels in the tested specimens. Due to the low proportion of culture confirmation, the majority of serotyping was performed on culture-negative or culture-not-performed specimens, which may have limited the ability to identify specific serotypes.

Because surveillance only captured meningitis cases, we were unable to estimate PCV13 impact on other clinical syndromes such as pneumonia and bacteremia, which may have a different serotype distribution. However, decreases in pneumococcal meningitis incidence among children aged <1 year observed in 2014 (49%) and 2015 (68%) were comparable to the decreases in IPD incidence reported in South Africa (31% among children aged <1 year by one year post-introduction) and The Gambia (55% among children aged <2 years by three years post-introduction).^14^, ^29^ Additionally, focusing exclusively on meningitis may preclude a full understanding of potential herd protection, as PCVs have been shown to decrease pneumonia among older age groups.^13^ Burkina Faso introduced PCV13 into the routine immunization program without a catch-up campaign, so full herd effects may not be seen until more birth cohorts are vaccinated or a booster dose is added to the schedule.^30^ Monitoring changes in pneumococcal carriage and additional years of surveillance will aid our understanding of PCV13 impact and pneumococcal transmission dynamics.^25^

As this is an ecologic study, we cannot attribute all changes in pneumococcal meningitis incidence directly to PCV13. However, ecologic studies remain important for evaluation of vaccination programs with high coverage and potential herd protection. While meningitis incidence varied considerably from year to year, children aged <1 year were the only age group to experience a lower pneumococcal meningitis incidence in both years post-introduction as compared to the three prior years. Despite surveillance quality improvement over time, challenges regarding specimen transport and laboratory confirmation in Burkina Faso remain; however, reported incidence rates adjust for changes in culture and rt-PCR testing capacity over time and likely reflect true trends in incidence.

This analysis adds to the literature regarding PCV13 impact in West Africa^14^ using nationwide population-based data and routine pneumococcal serotyping. Continued vigilant surveillance and serotyping in Burkina Faso is crucial to monitor medium- and long-term impacts of PCV13, including changes in incidence among young children, herd protection among older age groups, impact on serotype 1 disease, and potential serotype replacement. Surveillance is also needed to rapidly detect any pneumococcal meningitis outbreaks, such as the serotype 1 outbreak experienced in Ghana in 2015–2016,^24^ which occurred among children aged ≥5 years despite two years of routine infant PCV13 immunization. This early evaluation of PCV13 impact shows encouraging results, and if trends continue, PCV13 may build on the success of Hib vaccine and MenAfriVac^TM^ in reducing the burden of bacterial meningitis in Burkina Faso.

## Individual authors contributions

DK, HMS, ROT, IM, LM, RTN, and CVB conceptualized and designed the study. FA, CGW, RTN, and CVB assisted with funding acquisition. IY and GS collected surveillance data. DK, HMS, and GS cleaned the surveillance data. DK, ROT, LS, ASO, SO, MCO, and AKB conducted laboratory analyses. LM and VS reviewed and validated the laboratory analysis. HMS did the statistical analysis. DK and HMS drafted the manuscript. All authors assisted with critical review of the manuscript. CGW and CVB provided expert opinion on study design, reviewed the manuscript, and critically analyzed results.

## Declaration of interests

The authors have no conflicts of interest.

## Funding source

This work was supported by the MenAfriNet Consortium (www.menafrinet.org), a partnership between the U.S. Centers for Disease Control and Prevention, World Health Organization, and Agence de Médecine Préventive, through a grant from the Bill and Melinda Gates Foundation (OPP1084298), and a grant from Gavi.
