# Supplementary material for: Early impact of 13-valent pneumococcal conjugate vaccine on pneumococcal meningitis—Burkina Faso, 2014–2015
Source: J Infect. 2018 Mar;76(3):270–9. doi: 10.1016/j.jinf.2017.12.002 (PMC5821694; doi:10.1016/j.jinf.2017.12.002)
Supplement: Appendix S1 — Supplementary Figures S1–S4 and Tables S1–S5. [file mmc1.docx]

**SUPPLMENTAL TABLES AND FIGURES**

**Supplemental Table 1. National meningitis data quality and completeness indicators, Burkina Faso, 2011–2015**

|  | **2011** | | | **2012** | | **2013** | | **Pre-PCV13**  **2011–2013** | | **2014** | | | **2015** | | **Post-PCV13**  **2014–2015** | | **Total 2011–2015** | | |  |
| --- | --- | --- | --- | --- | --- | --- | --- | --- | --- | --- | --- | --- | --- | --- | --- | --- | --- | --- | --- | --- |
|  | **N(%)** | | | **N(%)** | | **N(%)** | | **N(%)** | | **N(%)** | | | **N(%)** | | **N(%)** | | **N(%)** | | |  |
| Districts submitting case-based surveillance data | 57/63 | (90) | 63/63 | | (100) | 63/63 | (100) | 63/63 | (100) | 63/63 | (100) | 63/63 | | (100) | 63/63 | (100) | 63/63 | (100) | |  |
| Districts submitting CSF specimens | 57/63 | (90) | 63/63 | | (100) | 63/63 | (100) | 63/63 | (100) | 63/63 | (100) | 63/63 | | (100) | 63/63 | (100) | 63/63 | | (100) | |
| Suspected meningitis cases | 2,841 |  | 6,499 | |  | 2,829 |  | 12,169 |  | 3,399 |  | 2,970 | |  | 6,369 |  | 18,538 | |  | |
| Suspected meningitis cases with a LP^a^ | 2,766 | (97) | 6,302 | | (97) | 2,758 | (97) | 11,826 | (97) | 3,331 | (98) | 2,912 | | (98) | 6,243 | (98) | 18,069 | | (97) | |
| CSFs assessed for appearance | 2,417 | (87) | 5,723 | | (91) | 2,585 | (94) | 10,725 | (91) | 3,129 | (94) | 2,711 | | (93) | 5,840 | (94) | 16,565 | | (92) | |
| CSFs with a Gram stain^b^ | 2,508 | (91) | 5,626 | | (89) | 2,460 | (89) | 10,594 | (90) | 2,949 | (89) | 2,686 | | (92) | 5,635 | (90) | 16,229 | | (90) | |
| CSFs tested by cytology | 1217 | (44) | 2,473 | | (39) | 1,271 | (46) | 4,961 | (42) | 1,586 | (48) | 1,362 | | (47) | 2,948 | (47) | 7,909 | | (44) | |
| CSFs tested at a national laboratory^c,d^ | 1,242 | (45) | 2,379 | | (38) | 1,836 | (67) | 5,457 | (46) | 2,039 | (61) | 2,166 | | (74) | 4,205 | (67) | 9,662 | | (53) | |
| CSFs tested by latex | 1,146 | (41) | 1,205 | | (19) | 513 | (19) | 2,864 | (24) | 716 | (22) | 308 | | (11) | 1,024 | (16) | 3,888 | | (22) | |
| CSFs tested by culture | 722 | (26) | 1,552 | | (25) | 637 | (23) | 2,911 | (25) | 563 | (17)^d^ | 420 | | (14)^e^ | 983 | (16)^e^ | 3,894 | | (22) | |
| CSFs cultured but found to be contaminated^f^ | 190/722 | (26) | 275/1,552 | | (18) | 107/637 | (17) | 572/2,911 | (20) | 123/563 | (22) | 98/420 | | (23) | 221/983 | (22) | 793/3,101 | | (20) | |
| CSFs tested by rt-PCR | 1,133 | (41) | 1,396 | | (22) | 1,704 | (62) | 4,233 | (36) | 1,958 | (59) | 2,115 | | (73) | 4,073 | (65) | 8,306 | | (46) | |
| CSFs tested by latex, culture or rt-PCR | 1,708 | (62) | 2,821 | | (45) | 2,005 | (73) | 6,534 | (55) | 2,302 | (69) | 2,224 | | (76) | 4,526 | (73) | 11,060 | | (61) | |

Abbreviations: CSF, cerebrospinal fluid; LP, lumbar puncture; rt-PCR, real-time polymerase chain reaction.

^a^ 1,502 (8%) of CSFs were tested by latex only, 829 (5%) by culture only, 4,778 (26%) by rt-PCR only, 527 (3%) by latex and culture, 990 (5%) by latex and rt-PCR, 1,669 (9%) by culture and rt-PCR, and 869 (5%) by all three methods. 6,907 (38%) were not tested by any of the three methods.

^b^ Possible reasons why a CSF specimen may not reach a district laboratory for gram staining: difficulty with specimen transport, geographic inaccessibility of certain health centers, insufficient transport media.

^c^ Defined as being tested by culture and/or rt-PCR.

^d^ Possible reasons why a CSF specimen may not reach or be tested via culture or rt-PCR at a national reference laboratory: insufficient transport media, insufficient quantity of CSF that was entirely used for Gram staining and cytology, non-viable or contaminated specimen.

^e^ There were stock-outs of trans-isolate medium in 2014–2015, which limited culture capacity.

^f^ 647 (82%) of CSFs cultured but found to be contaminated were also tested by rt-PCR.

**Supplemental Table 2. *S. pneumoniae* diagnostic test results among confirmed^a^ pneumococcal meningitis cases, Burkina Faso, 2014–2015**

|  | **No. positive for *S. pneumoniae* using method(s) (%)** | | | | | |  |
| --- | --- | --- | --- | --- | --- | --- | --- |
| **Diagnostic testing method** | **2014** | | **2015** | | **Total** | | |
| Latex only | 51 | (10·2) | 28 | (5·1) | 79 | (7·5) | |
| Culture only | 3 | (0·6) | 2 | (0·4) | 5 | (0·5) | |
| rt-PCR only | 308 | (61·4) | 444 | (80·6) | 752 | (71·4) | |
| Latex and culture only | 1 | (0·2) | 1 | (0·2) | 2 | (0·2) | |
| Latex and rt-PCR only | 117 | (23·3) | 59 | (10·7) | 176 | (16·7) | |
| Culture and rt-PCR | 10 | (2·0) | 11 | (2·0) | 21 | (2·0) | |
| Latex, culture and rt-PCR | 12 | (2·4) | 6 | (1·1) | 18 | (1·7) | |
|  | **No. positive / No. of confirmed^a^ cases tested using method (%)** | | | | | | |
| Latex | 181/207 | (87·4) | 94/111 | (84·7) | 275/318 | (86·5) | |
| Culture | 26/111 | (23·4) | 20/93 | (21·5) | 46/204 | (22·6) | |
| rt-PCR | 447/456 | (98·0) | 520/528 | (98·5) | 967/984 | (98·3) | |
| **Total positive for *S. pneumoniae*** | **502** |  | **551** |  | **1,053** |  | |

Abbreviations : rt-PCR, real-time polymerase chain reaction.

^a^ *S. pneumoniae* isolated from cerebrospinal fluid (CSF) by culture or detected in CSF by rt-PCR or latex. Not all specimens were tested via all three methods.

**Supplemental Table 3. Distribution of pneumococcal serotypes, Burkina Faso, 2011–2015**

| **Pneumococcal serotype** | **2011**  **N (%)** | | **2012**  **N (%)** | | **2013**  **N (%)** | | **Total**  **2011-2013**  **N (%)** | | **2014**  **N (%)** | | **2015**  **N (%)** | | **Total**  **2014-2015**  **N (%)** | |
| --- | --- | --- | --- | --- | --- | --- | --- | --- | --- | --- | --- | --- | --- | --- |
| ***PCV13 serotypes*** | ***291*** | ***(76)*** | ***234*** | ***(72)*** | ***212*** | ***(65)*** | ***737*** | ***(71)*** | ***298*** | ***(76)*** | ***340*** | ***(70)*** | ***638*** | ***(73)*** |
| 1 | 211 | (55) | 135 | (41) | 118 | (36) | 464 | (45) | 207 | (53) | 281 | (58) | 488 | (56) |
| 3 | 1 | (0·3) | 2 | (1) | 2 | (1) | 5 | (0·5) | 3 | (1) | 8 | (2) | 11 | (1) |
| 4 | 5 | (1) | 2 | (1) | 3 | (1) | 10 | (1) | 6 | (2) | 2 | (0·4) | 8 | (1) |
| 5 | 35 | (9) | 21 | (6) | 15 | (5) | 71 | (7) | 15 | (4) | 11 | (2) | 26 | (3) |
| 6A/6B | 8 | (2) | 19 | (6) | 19 | (6) | 46 | (4) | 21 | (5) | 4 | (1) | 25 | (3) |
| 7F/7A | 4 | (1) | 5 | (2) | 7 | (2) | 16 | (2) | 1 | (0·3) | 5 | (1) | 6 | (1) |
| 9V/9A | 0 | (0) | 2 | (1) | 1 | (0·3) | 3 | (0·3) | 2 | (1) | 5 | (1) | 7 | (1) |
| 14 | 16 | (4) | 7 | (2) | 11 | (3) | 34 | (3) | 11 | (3) | 4 | (1) | 15 | (2) |
| 18C/18F/18B/18A | 2 | (1) | 12 | (4) | 6 | (2) | 20 | (2) | 8 | (2) | 0 | (0) | 8 | (1) |
| 19A | 1 | (0·3) | 5 | (2) | 3 | (1) | 9 | (1) | 0 | (0) | 3 | (1) | 3 | (0·3) |
| 19F | 2 | (1) | 3 | (1) | 6 | (2) | 11 | (1) | 9 | (2) | 2 | (0·4) | 11 | (1) |
| 23F | 30 | (2) | 21 | (6) | 21 | (6) | 48 | (5) | 15 | (4) | 15 | (3) | 30 | (3) |
| ***Non-PCV13 serotypes*** | ***29*** | ***(8)*** | ***49*** | ***(15)*** | ***62*** | ***(19)*** | ***141*** | ***(14)*** | ***53*** | ***(14)*** | ***47*** | ***(10)*** | ***100*** | ***(11)*** |
| 2 | 3 | (1) | 8 | (2) | 6 | (2) | 17 | (2) | 5 | (1) | 5 | (1) | 10 | (1) |
| 7C/7B/40 | 0 | (0) | 0 | (0) | 0 | (0) | 0 | (0) | 1 | (0·3) | 0 | (0) | 1 | (0·1) |
| 8 | 0 | (0) | 0 | (0) | 2 | (1) | 2 | (0·2) | 0 | (0) | 0 | (0) | 0 | (0) |
| 9N/9L | 0 | (0) | 2 | (1) | 2 | (1) | 4 | (0·4) | 3 | (1) | 0 | (0) | 3 | (0·3) |
| 10A | 0 | (0) | 0 | (0) | 0 | (0) | 0 | (0) | 0 | (0) | 1 | (0·2) | 1 | (0·1) |
| 10F/10C/33C | 0 | (0) | 0 | (0) | 1 | (0·3) | 1 | (0·1) | 0 | (0) | 1 | (0·2) | 1 | (0·1) |
| 11A/11D | 1 | (0·3) | 0 | (0) | 0 | (0) | 1 | (0·1) | 4 | (1) | 2 | (0·4) | 6 | (1) |
| 12F/12A/12B/44/46 | 20 | (5) | 26 | (8) | 36 | (11) | 82 | (8) | 36 | (9) | 26 | (5) | 62 | (7) |
| 13 | 0 | (0) | 1 | (0·3) | 0 | (0) | 1 | (0·1) | 0 | (0) | 1 | (0·2) | 1 | (0·1) |
| 15A/15F | 0 | (0) | 0 | (0) | 0 | (0) | 0 | (0) | 0 | (0) | 1 | (0·2) | 1 | (0·1) |
| 15B/15C | 3 | (1) | 0 | (0) | 2 | (1) | 5 | (0·5) | 1 | (0·3) | 1 | (0·2) | 2 | (0·2) |
| 16F | 0 | (0) | 2 | (1) | 2 | (1) | 4 | (0·4) | 0 | (0) | 1 | (0·2) | 1 | (0·1) |
| 21 | 1 | (0·3) | 1 | (0·3) | 0 | (0) | 2 | (0·2) | 0 | (0) | 0 | (0) | 0 | (0) |
| 22F/22A | 1 | (0·3) | 1 | (0·3) | 0 | (0) | 2 | (0·2) | 0 | (0) | 0 | (0) | 0 | (0) |
| 23B | 0 | (0) | 0 | (0) | 1 | (0·3) | 1 | (0·1) | 1 | (0·3) | 0 | (0) | 1 | (0·1) |
| 24F/24A/24B | 0 | (0) | 0 | (0) | 2 | (1) | 2 | (0·2) | 0 | (0) | 0 | (0) | 0 | (0) |
| 25F/25A/38 | 1 | (0·3) | 6 | (2) | 6 | (2) | 13 | (1) | 2 | (1) | 7 | (1) | 9 | (1) |
| 33F/33A/37 | 0 | (0) | 1 | (0·3) | 0 | (0) | 1 | (0·1) | 0 | (0) | 0 | (0) | 0 | (0) |
| 34 | 0 | (0) | 0 | (0) | 1 | (0·3) | 1 | (0·1) | 0 | (0) | 0 | (0) | 0 | (0) |
| 35B | 0 | (0) | 1 | (0·3) | 1 | (0·3) | 2 | (0·2) | 0 | (0) | 1 | (0·2) | 1 | (0·1) |
| ***Non-typeable*** | ***63*** | ***(16)*** | ***43*** | ***(13)*** | ***52*** | ***(16)*** | ***158*** | ***(15)*** | ***39*** | ***(10)*** | ***100*** | ***(21)*** | ***139*** | ***(16)*** |
| **Total serotyped** | **384** | **(60)** | **326** | **(71)** | **326** | **(77)** | **1,036** | **(68)** | **390** | **(78)** | **487** | **(88)** | **877** | **(83)** |
| **Missing serotype^a^** | **258** | **(40)** | **136** | **(29)** | **98** | **(23)** | **492** | **(32)** | **112** | **(22)** | **64** | **(12)** | **176** | **(17)** |
| **Total** | **642** |  | **462** |  | **424** |  | **1,528** |  | **502** |  | **551** |  | **1,053** |  |

^a^ 350 (14%) cases were only positive via latex and could not be serotyped: 151 from 2011, 83 from 2012, 38 from 2013, 50 from 2014, and 28 from 2015. Serotype results were unavailable for 355 culture- and/or rt-PCR-positive cases: 107 from 2011, 53 from 2012, 60 from 2013, 62 from 2014, and 73 from 2015.

**Supplemental Table 4. Pneumococcal meningitis cases among PCV13-eligible children, by vaccination status and serotype, Burkina Faso, 2013–2015**

| **PCV13 doses** | **PCV13 serotypes** | **Non-PCV13 serotypes** | **Missing serotype** | **Total** |
| --- | --- | --- | --- | --- |
| **0 doses** | **8** | **10** | **4** | **22** |
| Serotypes | *7F/7A (n=2), 14 (n=2), 1, 4, 6A/6B, 23F* | *NT (n=4), 12F/12A/12B/44/46 (n=3), 2 (n=2), 9N/9L* |  |  |
| **1 dose** | **7** | **4** | **1** | **12** |
| Serotypes | *1 (n=3), 5 (n=2), 6A/6B, 23F* | *NT (n=2), 12F/12A/12B/44/46 (n=2)* |  |  |
| **2 doses** | **2** | **8** | **4** | **14** |
| Serotypes | *1, 23F* | *NT (n=3), 12F/12A/12B/44/46 (n=2), 2, 10F/10C,33C, 11A/11D* |  |  |
| **3 doses** | **9** | **9** | **8** | **26** |
| Serotypes | *7F/7A (n=2), 14 (n=2), 1, 4, 6A/6B, 23F* | *NT (n=5), 12F/12A/12B/44/46 (n=2), 15B/15C, 35B* |  |  |
| **Unknown** | **2** | **1** | **2** | **5** |
| Serotypes | *6A/6B, 14* | *12F/12A/12B/44/46* |  |  |
| **Total** | **28** | **32** | **19** | **79** |

Abbreviations: PCV13, 13-valent pneumococcal conjugate vaccine.

**Supplemental Table 5. Pneumococcal meningitis cases among PCV13-eligible children, Burkina Faso, 2013–2015**

| **No. PCV13 doses ≥2 weeks prior to illness onset** | **Month/year of illness onset** | **Age (months) at illness onset** | **Months since last dose** | **PCV13 dose 1: Age (months)** | **PCV13 dose 2: Age (months)** | **PCV13 dose 3: Age (months)** | **Serotype** |
| --- | --- | --- | --- | --- | --- | --- | --- |
| ***PCV13-serotype cases*** | | | | | | | |
| 3 doses | 8/2014 | 7·9 | 4·0 | 2·0 | 2·9 | 3·8 | 1 |
|  | 11/2014 | 8·5 | 3·8 | 2·1 | 3·5 | 4·7 | 5 |
|  | 11/2014 | 15·3 | 6·3 | 5·4 | 7·0 | 9·0 | 6A/6B |
|  | 1/2015 | 12·0 | 7·9 | 2·2 | 3·2 | 4·1 | 23F |
|  | 2/2015 | 11·4 | 6·8 | 2·5 | 3·6 | 4·5 | 1 |
|  | 2/2015 | 6·0 | 1·9 | 2·1 | 3·2 | 4·1 | 23F |
|  | 3/2015 | 11·6 | 7·1 | 2·3 | 3·4 | 4·5 | 23F |
|  | 4/2015 | 12·1 | 7·7 | 2·2 | 3·2 | 4·4 | 23F |
|  | 7/2015 | 11·5 | 7·5 | 2·0 | 3·1 | 4·0 | 1 |
| 2 doses | 1/2014 | 4·1 | 1·2 | 1·6 | 2·9 |  | 23F |
|  | 4/2015 | 5·2 | 1·1 | 3·1 | 4·1 |  | 1 |
| 1 dose | 6/2014 | 3·1 | 0·9 | 2·2 |  |  | 5 |
|  | 11/2014 | 8·8 | 7·0 | 1·8 |  |  | 5 |
|  | 11/2014 | 10·3 | 8·4 | 1·8 |  |  | 23F |
|  | 11/2014 | 8·7 | 6·4 | 2·3 |  |  | 6A/6B |
|  | 12/2014 | 10·8 | 8·9 | 1·9 |  |  | 1 |
|  | 5/2015 | 6·4 | 2·5 | 4·0 |  |  | 1 |
|  | 10/2015 | 13·3 | 11·0 | 2·3 |  |  | 1 |
| 0 doses | 1/2014 | 3·8 |  |  |  |  | 4 |
|  | 3/2014 | 2·0 |  |  |  |  | 1 |
|  | 12/2014 | 3·3 |  |  |  |  | 14 |
|  | 2/2015 | 6·3 |  |  |  |  | 6A/6B |
|  | 3/2015 | 2·8 |  |  |  |  | 14 |
|  | 3/2015 | 2·2 |  |  |  |  | 7F/7A |
|  | 5/2015 | 2·2 |  |  |  |  | 7F/7A |
|  | 6/2015 | 4·1 |  |  |  |  | 23F |
| ***Non-PCV13-serotype cases*** | | | | | | | |
| 3 doses | 2/2014 | 4·7 | 0·5 | 2·0 | 3·2 | 4·3 | NT |
|  | 10/2014 | 12·8 | 9·0 | 2·0 | 3·0 | 3·9 | 12F/12A/12B/44/46 |
|  | 11/2014 | 14·8 | 10·3 | 2·6 | 3·5 | 4·5 | 15B/15C |
|  | 12/2014 | 12·0 | 6·8 | 2·2 | 3·2 | 5·2 | 12F/12A/12B/44/46 |
|  | 3/2015 | 7·3 | 3·1 | 2·2 | 3·2 | 4·3 | NT |
|  | 4/2015 | 5·7 | 1·7 | 2·0 | 3·0 | 4·1 | NT |
|  | 4/2015 | 9·8 | 4·6 | 3·4 | 4·3 | 5·2 | 35B |
|  | 6/2015 | 20·2 | 14·2 | 2·1 | 3·3 | 6·0 | NT |
|  | 8/2015 | 15·0 | 10·6 | 2·3 | 3·4 | 4·4 | NT |
| 2 doses | 11/2014 | 14·5 | 6·0 | 3·5 | 8·5 |  | 11A/11D |
|  | 11/2014 | 8·8 | 5·2 | 2·5 | 3·6 |  | NT |
|  | 12/2014 | 15·2 | 9·4 | 4·9 | 5·9 |  | 12F/12A/12B/44/46 |
|  | 12/2014 | 11·2 | 6·9 | 3·5 | 4·3 |  | 2 |
|  | 12/2014 | 15·2 | 12·2 | 2·1 | 3·0 |  | 10F/10C/33C |
|  | 12/2014 | 5·9 | 2·4 | 2·1 | 3·5 |  | 12F/12A/12B/44/46 |
|  | 3/2015 | 4·6 | 0·6 | 2·6 | 3·9 |  | NT |
|  | 4/2015 | 7·8 | 3·4 | 2·2 | 4·4 | 10·6 | NT |
| 1 dose | 4/2014 | 3·5 | 1·2 | 2·3 | 3·3 | 4·2 | NT |
|  | 12/2014 | 11·7 | 7·8 | 3·8 |  |  | 12F/12A/12B/44/46 |
|  | 1/2015 | 3·7 | 1·4 | 2·3 | 3·4 |  | NT |
|  | 2/2015 | 3·3 | 1·2 | 2·1 |  |  | 12F/12A/12B/44/46 |
|  | 3/2014 | 5·0 |  |  |  |  | 12F/12A/12B/44/46 |
|  | 3/2014 | 6·3 |  |  |  |  | 9N/9L |
|  | 4/2014 | 2·6 |  | 3·6 | 4·7 | 5·6 | NT |
|  | 11/2014 | 10·5 |  |  |  |  | 12F/12A/12B/44/46 |
|  | 1/2015 | 2·3 |  | 2·0 | 3·1 | 4·0 | NT |
|  | 2/2015 | 2·2 |  | 3·2 | 3·9 | 5·0 | 2 |
|  | 3/2015 | 5·2 |  |  |  |  | 12F/12A/12B/44/46 |
|  | 4/2015 | 3·2 |  |  |  |  | NT |
|  | 4/2015 | 2·4 |  | 2·1 |  |  | NT |
|  | 6/2015 | 2·0 |  | 2·0 | 3·1 | 4·3 | 2 |

*
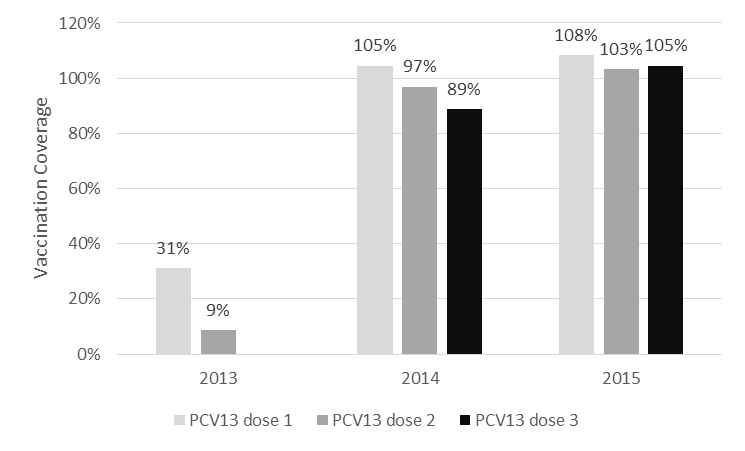
*

**Supplemental Figure 1.** Cumulative administrative 13-valent pneumococcal conjugate vaccine (PCV13) coverage among children aged 0–11 months, Burkina Faso, November 2013–December 2015


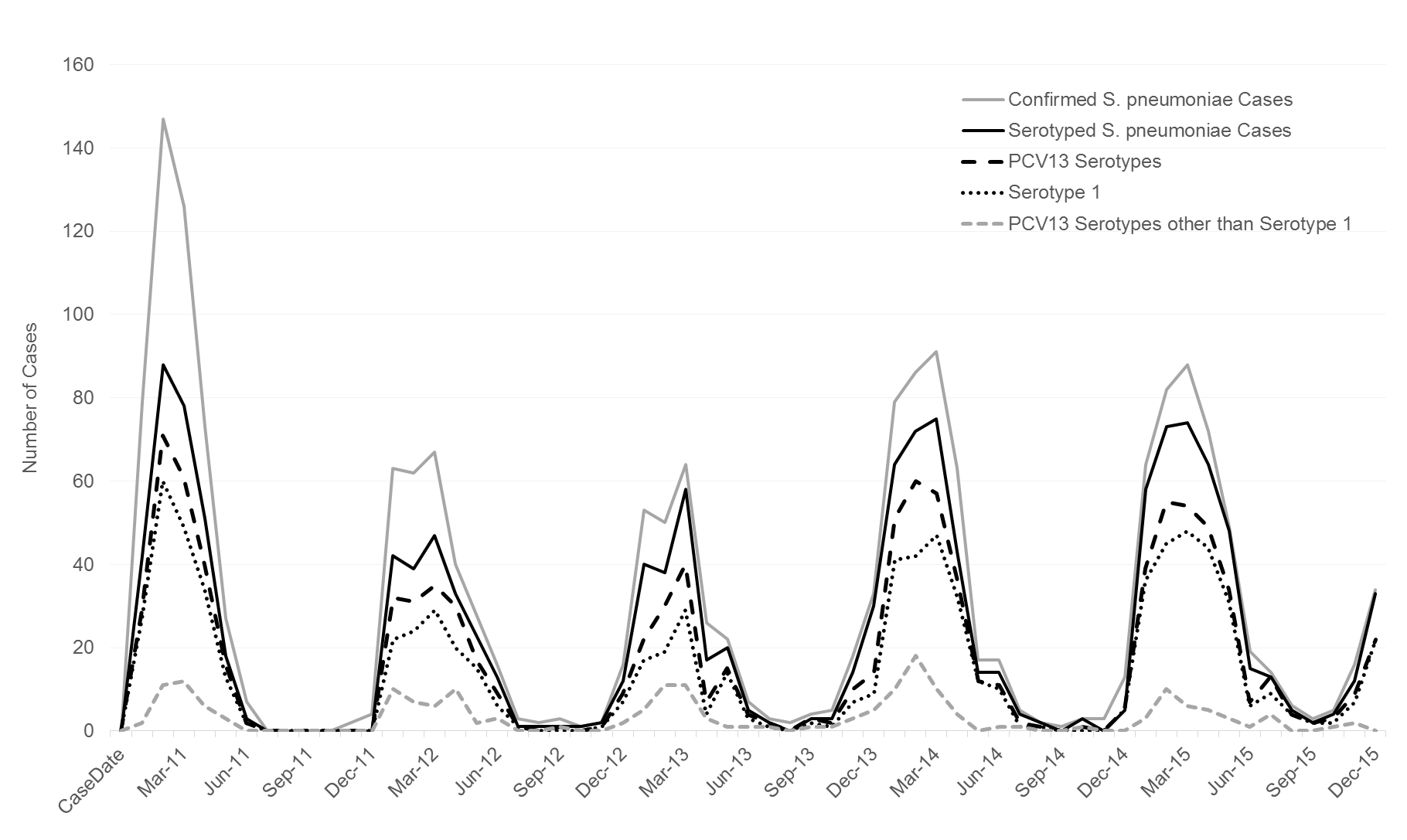


**Supplemental Figure 2.** Epidemic curve of confirmed* pneumococcal meningitis cases, serotyped cases, PCV13 serotypes, serotype 1, and PCV13 serotypes other than serotype 1 among persons aged ≥5 years, by month, Burkina Faso, 2011–2015. **S. pneumoniae* isolated from cerebrospinal fluid (CSF) by culture or detected in CSF by real-time polymerase chain reaction or latex.

**
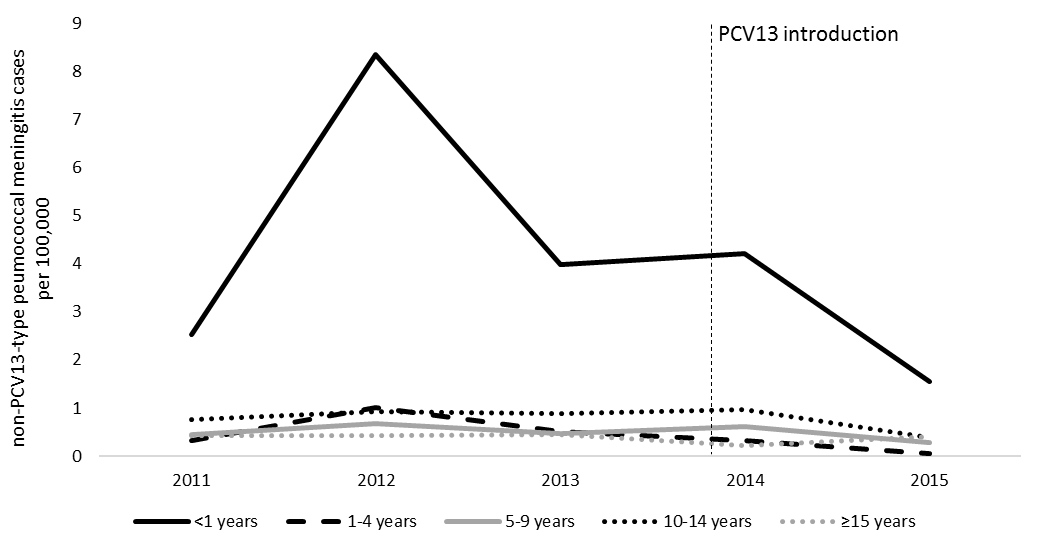
**

**Supplemental Figure 3.** Incidence of pneumococcal meningitis caused by non-PCV13 serotypes, by year and age group, Burkina Faso, 2011–2015

**
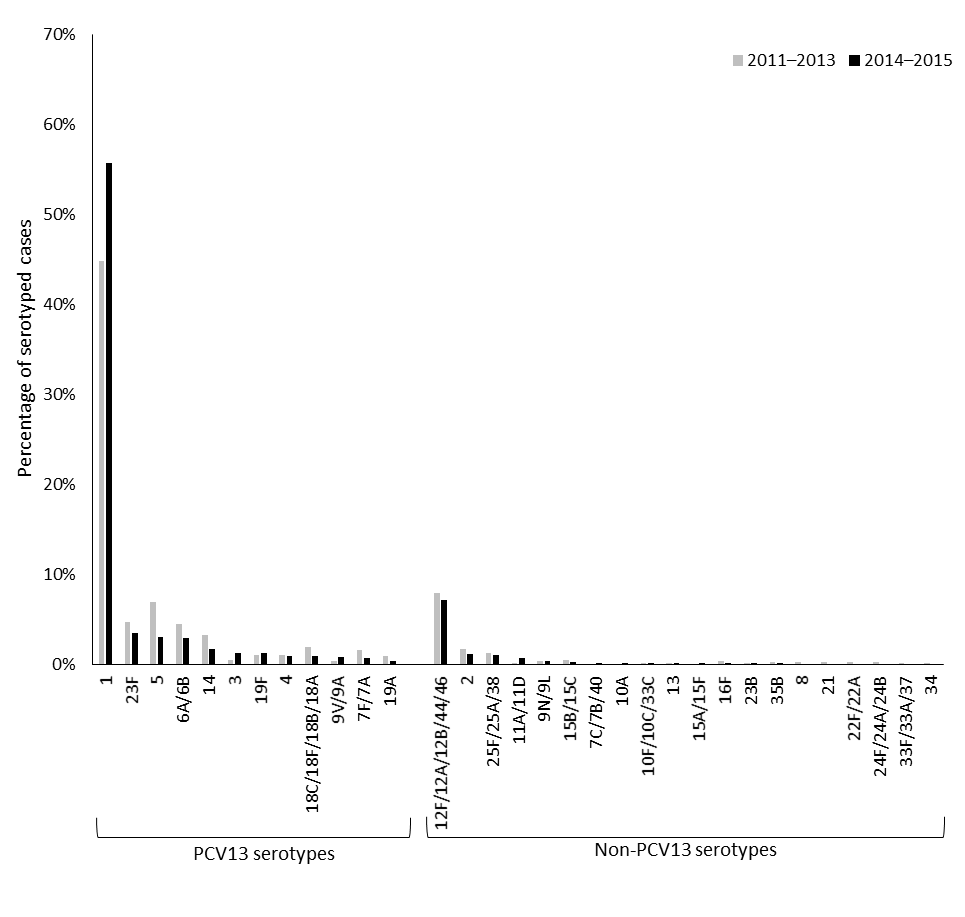
**

**Supplemental Figure 4.** Percentage of serotyped pneumococcal meningitis cases due to each serotype among all ages in 2011–2013 vs. 2014–2015, Burkina Faso. Non-typeable serotypes are not shown in this figure.
